# Energy efficiency as a unifying principle for human, environmental, and global health

**DOI:** 10.12688/f1000research.2-101.v1

**Published:** 2013-04-02

**Authors:** Luigi Fontana, Vincenzo Atella, Daniel M Kammen

**Affiliations:** 1 Division of Geriatrics and Nutritional Science, Washington University School of Medicine, St. Louis, MO, 63110, USA; 2 Department of Medicine, Salerno University Medical School, Salerno, 84081, Italy; 3 CEINGE Biotecnologie Avanzate, Napoli, 80145, Italy; 4 Department of Economics and Finance, University of Rome Tor Vergata, Rome, 00133, Italy; 5 Center for Health Policy, Stanford University, Stanford, CA, 94305-6019, USA; 6 Energy and Resources Group, University of California, Berkeley, Berkeley, CA, 94720–3050, USA; 7 Goldman School of Public Policy, University of California, Berkeley, Berkeley, CA, 94720–3050, USA; 8 Renewable and Appropriate Energy Laboratory, University of California, Berkeley, Berkeley, CA, 94720–3050, USA

## Abstract

A strong analogy exists between over/under consumption of energy at the level of the human body and of the industrial metabolism of humanity. Both forms of energy consumption have profound implications for human, environmental, and global health. Globally, excessive fossil-fuel consumption, and individually, excessive food energy consumption are both responsible for a series of interrelated detrimental effects, including global warming, extreme weather conditions, damage to ecosystems, loss of biodiversity, widespread pollution, obesity, cancer, chronic respiratory disease, and other lethal chronic diseases. In contrast, data show that the efficient use of energy—in the form of food as well as fossil fuels and other resources—is vital for promoting human, environmental, and planetary health and sustainable economic development. While it is not new to highlight how efficient use of energy and food can address some of the key problems our world is facing, little research and no unifying framework exists to harmonize these concepts of sustainable system management across diverse scientific fields into a single theoretical body. Insights beyond reductionist views of efficiency are needed to encourage integrated changes in the use of the world’s natural resources, with the aim of achieving a wiser use of energy, better farming systems, and healthier dietary habits. This perspective highlights a range of scientific-based opportunities for cost-effective pro-growth and pro-health policies while using less energy and natural resources.

## Introduction

Several interrelated challenges now face the world, including (1) providing adequate food, clean drinking water, and non-renewable energy resources, which exist in finite supplies, to an exponentially growing population; (2) creating a sustainable global economy that does not destroy the environment or compromise human health; and (3) limiting the detrimental socioeconomic and health effects of the worldwide epidemic of unhealthy nutrition and obesity. How can we handle these challenges? We believe that today the right answer to many of these problems is a more efficient and wise use of energy, food, water, and other natural resources. The current economic model is unsustainable. As we will discuss in this paper, reliance on technology to produce more food and energy to drive economic growth can be successful in the short term, but it has long-lasting, seriously detrimental consequences for human and environmental health and, eventually, for societal well-being. In contrast, energy efficiency can play a key role in promoting human, environmental, and planetary health and sustainable economic development.

The term “energy efficiency” usually refers to devices or engineered systems that provide the same level of output or benefit with less energy consumption. However, accumulating scientific evidence indicates that energy efficiency is also an important principle for optimizing physiological functions within organisms, both simple and complex, including mammals. Hundreds of studies have shown that moderately energy-restricted animals live much longer and in better health than animals that have free access to food energy ^1– 3^. A similar outcome is seen when the activity of energy-sensing cellular pathways (i.e. the insulin and insulin-like growth factor 1 signaling pathway and the mammalian target of rapamycin pathway) is reduced by genetic manipulation or chemical inhibitors ^2^.

Similarly, and in a way that is not just analogical but organically and causally linked, energy and resource efficiency is vital at the level of society and the biosphere. Essential for achieving environmental health and sustainable economic development, reducing the consumption of non-renewable energy and other natural resources has profound implications for human health as well. While it is clear that a lack of access to energy and resources, or their uneven distribution, chokes economic development, excess energy consumption from fossil-fuel sources promotes extensive pollution and global warming, and is a sign of economic and public ill-health and inequality ^4^. Squandering energy resources, even if carbon-free, has collateral impacts–such as potential excessive exposure to cadmium or other toxic compounds, excessive mining and demand for rare earths and other precious materials, water depletion, and resource waste, which degrades well-being ^5^. More generally, energy and other resource waste is a critical sign of a system that is not providing for basic needs or supporting innovation, and is ultimately damaging the biosphere and human health as well ^6^.

Correcting excessive dietary energy intake to achieve optimal body weight and health, and deploying more energy-efficient buildings, vehicles, appliances and industrial equipment, fit into a continuum of actions that hold the potential to reduce the world’s projected energy needs by more than half, and to become the prime movers of cost-effective control of pollution and global greenhouse gas emissions ^7, 8^. In contrast, producing and consuming more fossil-fuel and food energy causes a vicious cycle by increasing unhealthy emissions, global warming, floods, droughts, land desertification, water shortages, and ultimately, reduced crop harvests ^9, 10^.

We propose the concept that a deep parallel connection exists between over/under consumption of energy at the level of the human body and at the level of the biosphere, and that this connection has profound implications for human and environmental health. While it is not new to highlight how efficient use of energy and other resources can address some of the key problems our world is facing, little or no unifying framework exists to combine and harmonize these concepts of sustainable system management across diverse fields (i.e. biology, medicine, ecology, economics, engineering, information technology, etc.) into a single theoretical body. Clearly, there is a need for new strategies and effective policies that encourage integrated changes in the use of the world’s natural resources, with the aim of achieving a wiser use of energy, better farming systems, and healthier dietary habits. We believe it is necessary to develop more complex models of analysis based on a multi-objective set of constraints.

## Energy efficiency and human health

**Figure 1.**
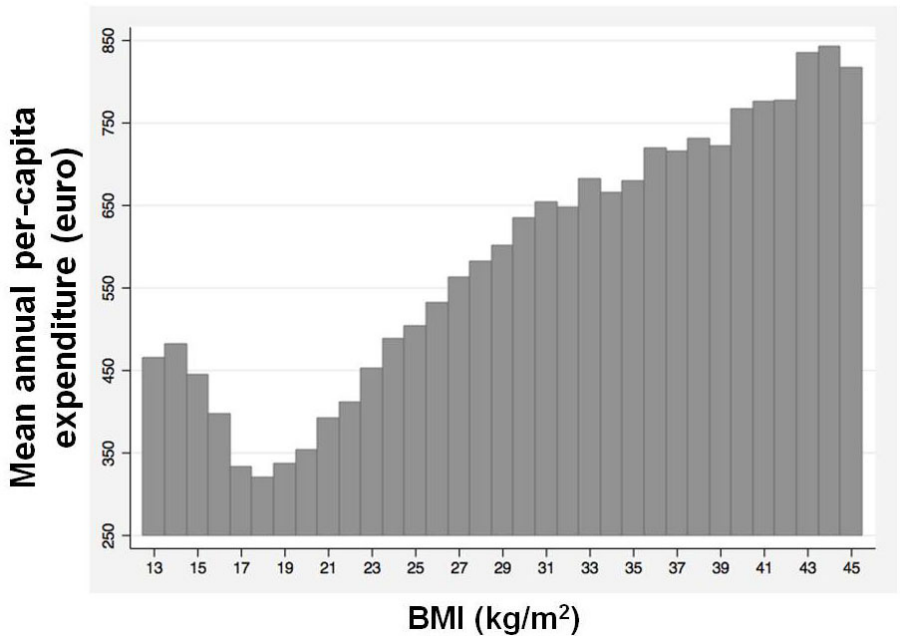
Average annual per capita health care cost by body mass index (BMI) categories. Age-adjusted outpatient health care costs (e.g. pharmaceutical, diagnostic and specialist visit expenditure) are shown per capita per year. We examined the relationship between BMI and medical care expenditure based on a sample of 423,682 Italian adults aged 18–95 in 2008–2010 (unpublished data).

Life expectancy at birth has almost doubled in most developed countries over the last century, with the oldest group (aged >65 years) being the most rapidly growing segment of the population ^11^. However, the overall increase in average life span is far greater than that of healthy lifespan. Globally, about 80% of older adults are affected by at least one chronic disease, and 50% have two or more chronic diseases (e.g. cardiovascular disease, stroke, cancer or type 2 diabetes) ^12^. These chronic diseases, which according to the World Health Organization (WHO) are largely preventable ^13^, are the leading cause of morbidity and mortality, as well as major contributors to economic losses and a driver of social burdens ^12, 13^. These problems are exacerbated by the current epidemic of excess weight and obesity, in which excessive adiposity is causally associated with an increased risk of developing type II diabetes, cardiovascular disease, cancer, and disability ^3, 12, 13^. Accordingly, our (unpublished) data, derived from a very large dataset of Italian patients seen by general practitioners through the National Health Search Network, show that excessive body weight is associated with a striking increase in health-care costs that could very likely lead to the bankruptcy of the health care system ( Figure 1). Clearly, Italy is not an exception in the industrialized world.

At the organismic level, sufficient but not excessive energy intake is vital for promoting health and longevity ^2^. At the extremes, both insufficient (i.e. starvation) and excessive (i.e. overweight and obesity) energy intake cause unfavorable alterations in body composition, metabolism, and organ function, eventually leading to premature death ^3^. In contrast, data from a multitude of studies indicate that a moderate reduction in energy intake below usual *ad libitum* levels without malnutrition prevents or delays a wide range of chronic diseases, results in a dramatic increase in healthspan and lifespan, and preserves a number of measured metabolic and physiologic functions found in experimental animals in more youthful-like states ^1– 3^. The beneficial effects of dietary restriction (DR) in rodents can be achieved by reducing energy consumption, but also by reducing protein or methionine intake ^1– 3, 14– 16^. These data have recently been confirmed in nonhuman primates. In a 20-year study, adult rhesus monkeys subjected to a 30% reduction in dietary intake experienced no diabetes, a 50% decline in cancer and cardiovascular morbidity and mortality, and less sarcopenia and neurodegeneration ^17, 18^.

Furthermore, data from recent clinical studies indicate that in humans, DR results in some of the same metabolic and physiologic adaptations related to healthy longevity found in DR rodents ^2, 3^. Individuals practicing moderate DR with adequate nutrition are powerfully protected against obesity, type 2 diabetes, high blood pressure, inflammation, and cardiovascular disease, and have lower cancer risk factors ^3, 19, 20^. Moreover, it has been shown that reducing dietary protein intake lowers the circulating levels of a key growth factor (IGF-1) that plays an important role in the pathogenesis of prostate, breast and colon cancer, and in the aging process itself ^21, 22^. This finding is important because the recommended daily allowance (RDA) for protein intake is 0.83 g/kg of body weight/day, yet at least 50% of men and women in many developed countries chronically consume twice as much protein as the recommended intake ^23^. Related to this problem is the fact that the majority of protein in the diet of North American and European citizens comes from foods of animal origin that promote weight gain and are rich in atherogenic saturated fatty acids ^24, 25^. Overconsumption of animal protein relative to other nutrients is not only a current epidemic among the affluent, but is a clear aspirational goal of millions of poor people in developing countries who are disproportionately increasing their consumption of meat and dairy as their wages rise above poverty level ^7^. The pressure of this overconsumption of animal foods on water and land use is intense, with 70% of all land under tillage used to feed livestock, which can have as high as a 21:1 ratio of vegetable input to meat output ^26^. By contrast, if global dietary patterns changed to reduce the consumption of animal source foods and led to the adoption of a diet rich in plant-based foods, only 30 to 40% of the crops cultivated currently would be needed, significantly reducing air, water and land pollution (from the intensive use of reactive nitrogen and phosphorus fertilizers, and pesticides, and the poor management of animal wastes in many regions) ^27– 29^, topsoil impoverishment, over-pumping of groundwater, agriculture-related fuel consumption, and greenhouse gas emissions ^30, 31^. Furthermore, if people ate fewer foods rich in empty calories, less meat- and dairy-derived foods and consumed more vegetables, fruits, beans, whole grains, seeds, and nuts, overweight and obesity rates could be reduced and many age-associated chronic diseases could be prevented, significantly reducing the gap between lifespan and healthspan, as well as health care costs ^3, 12, 13, 19, 24, 25^.

## Social and environmental consequences of energy inefficiency

According to the United Nations Population Division, the world’s population reached seven billion in 2012, and it is expected to reach nine billion by 2050 ^32^. This unprecedented population expansion will require a massive increase in energy and food production to meet increasing demands. Although, we have the technology to further increase energy and food production (e.g. off-shore oil drilling, hydraulic fracturing for extracting natural gas from shale rock layers, biofuels, use of new pesticides, antibiotics, and more chemical fertilizers, genetically modified crops, etc.), this escalating demand will collide with the finite planet’s natural resources and the capacity to further absorb the increasing emissions produced by billions of people who live and work in energy-inefficient buildings; drive energy-inefficient, polluting motor vehicles; and desire to consume the same unhealthy, high-calorie diets rich in animal protein and fat that are typical of Western countries.

Today, fossil fuels account for roughly 85% of total energy use worldwide for the heating/cooling of buildings, transportation, industrial activities, manufacturing, and other applications ^8, 33^. The use of this non-renewable energy mix is responsible for roughly 80% of the total anthropogenic greenhouse gas emissions, and for half of the short-lived greenhouse gases such as methane ^33^. It has been estimated that intensive agriculture and animal farming alone already contribute almost 20% of worldwide annual greenhouse gas emissions ^31, 33^, and are responsible for increasing soil erosion and water resource pollution and depletion ^34^. Soil erosion and water pollution associated with intensive farming in turn lead to even greater use of fossil fuels and more global warming, because more energy is needed to process polluted water and to produce more hydrocarbon-based fertilizers and pesticides in order to grow monoculture crops in a topsoil increasingly depleted of nutrients ^29, 31, 34^.

Global warming is now seen scientifically as a major threat to not only life but also livelihoods on our planet, and has serious environmental, social, and economic implications. Even the warming to which we are already committed in the coming decades (only a 1–2.5°C increase), is now predicted to have significant environmental consequences, including drought and land desertification, water shortages and reduced crop harvests, floods due to extreme weather and glacial retreat, inundation of coasts and small islands due to sea level rise, more frequent and devastating storms, extinction of plant and animal species, and diffusion of climate-sensitive diseases such as malaria ^9, 10^.

**Figure 2.**
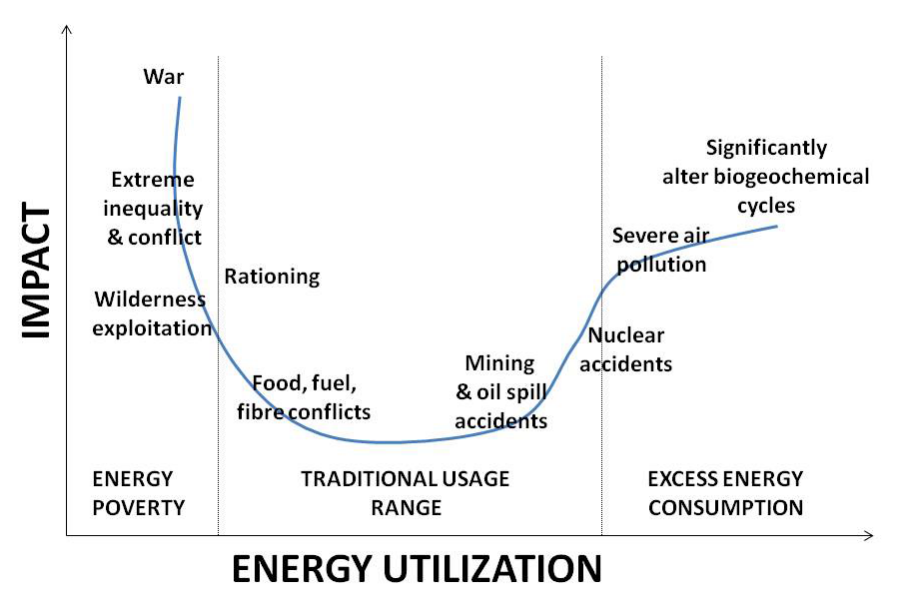
An energy sufficiency-efficiency cost curve. A schematic comparison of costs of energy poverty and excess energy consumption. The rough U-shape is characteristic of systems with excess impacts related to extremes of resource access and use. An associated aspect of the process of defining a regime of ‘wise use’ of resources is the role of efficiency relative to robustness of the system. A useful alternate representation is to place efficiency (“streamlining”) and diversity (“robustness”) as extremes on a single axis ^38^.

While climate change is the hot-button issue in our global energy metabolism, other byproducts generated by the excessive use of pesticides, chemical fertilizers, and antibiotics in intensive agriculture, the combustion of fossil fuels, the mining of coal and other metals, drilling for oil, and nuclear accidents are also direct causal agents of serious morbidity and mortality events for humans (e.g. cancer, chronic respiratory disease, asthma, and heart disease) and for the environment (e.g. acid rain and eutrophication resulting in toxic algal blooms, hypoxia, increased incidences of fish kills, loss of biodiversity, topsoil erosion, and water pollution) ^34– 36^. Figure 2 shows a range of impacts for insufficient and excessive energy resource access.

The resource efficiency paradigm has clear and immediate impacts when applied to the global energy budget. It has been estimated that the development and wide-spread use of more energy-efficient residential and industrial buildings, ultra-efficient lighting technologies, energy-saving appliances, and light-weight ultra-low-drag hybrid-electric motor vehicles could save 70% or more of the energy that we consume every day and drastically reduce CO _2_ emissions and pollution ^8, 33^. These steps are important individually, but take on particular significance when these patterns of highly-efficient use are taken as more than one-off policies, but as guiding principles for the design of the entire energy and natural resource utilization systems. Further, fossil fuel use via simple-cycle turbines is inherently wasteful due to the thermodynamic requirement to reject, or emit to the environment all energy beyond the Carnot efficiency limit of η = (total work/heat transferred from the engine) = 1 – (T _cold_/T _hot_), or roughly one third of the energy in the fuel for a standard power plant. This waste can be partially re-captured if overall system efficiency is taken as a design principle and a ‘combined heat and power’ system is utilized instead to capture the rejected heat for other uses such as warming homes or driving a second, lower energy engine cycle. Moreover, energy use of industrial equipment could be further reduced by changing their technical design, by using smart materials in conjunction with sensors and software that promote energy efficiency under all operating conditions. Finally, to improve energy resilience, it is necessary to combine energy efficiency with a steep increase in the development of renewable energy resources and the use of information and smart technology ^37^. Using digital intelligence and smart technologies to improve the current grid systems could prevent outages and faults, restore outages faster, and help manage demands. For example, by assessing energy needs through use of meters, sensors, digital controls and analytic tools to monitor, control and automate the two-way flow of energy across operations, energy consumption could be substantially reduced. Smart grids can also integrate renewable energy sources (i.e. solar, wind and geothermal power), and interact locally with distributed power sources, or plug-in electric vehicles.

## Conclusions

Significantly improving human and environmental health, societal wealth and well-being is possible, but requires a profound transformation in the way we live, and a new environment-centered industrial and economic system. Most of the needed knowledge and technology to enact a reshaping of our future and a new industrial revolution already exist today. In summary, we need to abandon the paradigm of producing more energy, food, and other products at lower cost in favor of a new paradigm that opts for less but high-quality energy, food and materials for a healthier life and environment. At the individual level, reducing the intake of calories by increasing the consumption of a variety of minimally processed plant foods and by significantly reducing the intake of animal foods will significantly increase health span and reduce health care costs, environmental pollution, soil erosion, water pollution and shortage, CO _2_ production and global warming, violent weather and associated planetary consequences. Similarly, making our houses more energy efficient and resilient (e.g. wall and roof insulation, energy-efficient windows and doors, ultra-efficient lighting technologies, energy-saving appliances, solar power to heat water and produce electricity, geothermal heat pumps, etc.), buying lightweight hybrid-electric motor vehicles, and reducing waste by choosing reusable products instead of disposables have huge effects in protecting human and environmental health as well. At the community level, we need more public/private investment and research in “green” chemistry, technologies and practices, including sustainable farming, breakthrough materials to improve building and vehicle efficiency, new technologies that better extract energy from renewable sources, hydrogen-fueled cars and buildings, and applications of modern information technology to maximize energy efficiency and resilience. The application of the energy efficiency and resource productivity paradigm offers a new ground for business invention, sustainable growth and economic development.

We also need to design and implement policies that enhance literacy about human and environmental health; improve the livability of our cities and towns by implementing, for example, projects for non-motorized transport, green spaces and parks; reward good behaviors, while enforcing the true costs of poor behavior (e.g. by lowering health insurance premiums for people with healthy lifestyles and metabolic profiles, taxing carbon and junk food, and ending subsidies for mining, oil, coal, corn, soy, and intensive factory animal farming).

Most importantly, we need to understand that both individual and societal wealth, happiness, and well-being do not depend merely on the acquisition of material goods and on economic growth, but are powered by our physical and psychological health, the quality of life and the richness of our social relationships, and foremost by the health of the environment that supports all life on earth, our “natural capital” that must be preserved.
