# Supplementary material for: Energy efficiency as a unifying principle for human, environmental, and global health
Source: F1000Res. 2013 Apr 2;2:101. [Version 1] doi: 10.12688/f1000research.2-101.v1 (PMC3869478; doi:10.12688/f1000research.2-101.v1)
Supplement: Peer review history

# Peer review: Referee response for version 1

**Published:** 2013-08-06

**Authors:** Tony McMichael

**Affiliations:** 1 College of Medicine, Population and Environment, Australian National University, Acton, Australia

This paper provides an important, big-picture, integration of the fundamental role of energy efficiency in the metabolism of organisms and of human societies - a role that provides guidance towards achieving balance and stability in complex systems, be they human bodies or energy-dependent economies. Energy flow and conservation is the currency of the world's processes of neg-entropy at all scales - the counter to the universal intrinsic tendency of all ordered systems to degrade and become disordered, as energy degrades to heat. The authors have been thorough in their review and consideration of the relevant published literature from different fields of inquiry, and offer a unifying perspective on the achievement of stability, balance and sustainability.

I have read this submission. I believe that I have an appropriate level of expertise to confirm that it is of an acceptable scientific standard.

# Peer review: Referee response for version 1

**Published:** 2013-08-05

**Authors:** Riccardo Pietrabissa

**Affiliations:** 1 Electronics, Information and Bioengineering, University Politecnico di Milano, Milan, Italy

This paper, an opinion article, points out a parallelism between systems (human body, environment and industry, earth) using energy consumption as a criteria for comparison. The paper does not introduce significant new knowledge on each of these systems and their relations with energy consumption, but expresses a global frame to rethink the use of energy. It may concern the goal of optimization in contrast with maximization or minimization, quality with respect to quantity.  

The consequence of these considerations are in wiser design of processes: prevention for health, efficiency for devices, integration for more complex systems as factories, buildings, crops, transportation. The considerations stated by authors address some political issues that require deeper analyses, certainly not possible in this short paper. Among these: is it possible to face the healthy aging and environment/industry sustainability jointly by public and private? Is it convenient to prevent diseases rather then treat them? Will it be possible, useful and profitable to continue to waste energy in the future as we have done in the past? 

It could be trivial to discuss all those questions and many others, each is well known. The parallelism introduced by this paper allows a greater vision on the problem of the quality of life in a better world not only for us in the remaining years of life, but for the future generations. The opinion of the authors is an interesting multidisciplinary viewpoint that can open a discussion on the role of science in suggesting integrated solutions for those open questions.

I have read this submission. I believe that I have an appropriate level of expertise to confirm that it is of an acceptable scientific standard.

# Peer review: Referee response for version 1

**Published:** 2013-05-07

**Authors:** Paul Terry, Jiangang Chen

**Affiliations:** 1 Department of Public Health, University of Tennessee, Knoxville, TN, USA

People can consume calories beyond their need and gain unhealthy weight. In ways that are somewhat analogous, societies can consume fossil fuel and other resources inefficiently, thereby transforming and polluting the environment in ways that are harmful. If this were the main point of the article, the reader might feel that the message is too obvious, the supporting facts too well known. Rather, the main thrust of the article is to delineate those analogous connections. Faithful to the article’s title, the authors assert: “A strong analogy exists between over/under consumption of energy at the level of the human body and the industrial metabolism of humanity.” They continue: “Similarly [with respect to the activity of energy-sensing cellular pathways in people], in a way that is not just analogical but organically and causally linked, energy and resource efficiency is vital at the level of society and the biosphere… We propose the concept that a deep parallel connection exists between over/under consumption of energy at the level of the human body and at the level of the biosphere, and that this connection has profound implications for human and environmental health.”  

Here, we were unsure as to the exact nature of their thesis, but it seemed the authors may be suggesting a remarkable synergy of very different effects on health, which may be measurable and ultimately help to define research priorities and improve study methods. However, the next two sections in the manuscript discuss the human health consequences of dietary energy over-consumption in individuals, and industrial over-consumption in societies, respectively, without showing many, if any, clear parallels. For example, it may be true that replacing meat consumption with that of vegetables may benefit both individuals and the environment, but clearly through different mechanisms and with different arrays of outcomes. This latter is a problem the authors don’t acknowledge. And, of course, under-consumption has very different implications to individuals, their cells, society and the environment, but except for the inclusion of the term in the sentences quoted above, under-consumption and its various implications are not discussed.  In the previous examples and others, the “deep parallel connections” between cells and biosphere were neither self-evident nor made clear by the authors. Consequently, much of this opinion piece on energy consumption, particularly as expressed by the authors as an analogical link between human cells, society, and the biosphere, was not convincing. We were left with the obvious fact that over-consumption of personal or industrial fuel has consequences to human health.  

In summary, we found the main motif of the article, namely, the analogical linkage between personal and societal energy consumption, lacking in clarity, structure, and careful support. Data presented in tables lacked description of source and methods, making them difficult for us to interpret as well. Even so, we commend the authors for expressing poignant and relevant thoughts about how we as individuals, and as members of a society, may protect our future health, and how individual lifestyle may interact with the environment in that regard.

We have read this submission. We believe that we have an appropriate level of expertise to confirm that it is of an acceptable scientific standard, however we have significant reservations, as outlined above.

# Response

**Published:** 2013-05-13

**Authors:** Luigi Fontana

**Affiliations:** Center for Human Nutrition, Washington University School of Medicine, USA

This is an opinion article, and not a research or a systematic review article, in which a physician scientist expert of human nutrition and longevity, an engineer expert in energy efficiency, and an economist expert in health economics, joined in an interdisciplinary manner to highlight some of the main interconnected problems that our world is facing. We believe that the reviewers missed the aim of our “opinion” paper, and did not make any effort to read some of the reference articles that have been cited to support our thoughts. 

It is impossible, in twenty-eight hundred words, to present and discuss in detail all the topics that we have addressed in this paper. Our main aim was just to combine research data and concepts generated from our and other laboratories in order to stimulate the discussion and stress the importance of an interdisciplinary approach to address many of the problems that are negatively impacting human and environmental health around the developed and developing world.    

It is not true that we discussed the consequences of dietary energy over-consumption in individuals, but instead we discussed the health consequences of calorie and protein restriction without malnutrition on metabolic health. We highlighted the importance of a new set of data on the molecular mechanisms regulating healthy aging and longevity, and the importance of these data in redesigning the health and agriculture systems. 

We highlighted how new scientific data generated from metabolic and molecular studies on animal model of longevity and humans support the concept that less calories and proteins (especially animal proteins) are needed to promote human and environmental health and to reduce global warming. 

To our knowledge, nobody working in the field of sustainable agriculture and environmental health has ever attempted to link the molecular mechanisms regulating healthy longevity (i.e. down regulation of the cellular energy-sensing pathways by calorie and protein restriction) with environmental health. We also tried to highlight how ancestral cellular and molecular mechanisms have been designed to promote health when food is scarce, and energy is used efficiently not for growth but for cell repair and maintenance. Analogically, we stressed how energy efficiency, and not more production of food and energy, is essential to promote both environmental and human health, and sustainable economic development. 

In the medicine field, most of the research funding is devoted to study drugs that may block pathways that are promoting cancer, cardiovascular disease, type 2 diabetes, and other chronic disease. In contrast, a trivial amount of research money is spent to study and implement dietary interventions that promote healthy longevity. In the meantime, for example in the United States, an alarming 70% and 33% of the adults are overweight or obese, respectively. Moreover, only approximately 7% of the remaining normal weight (BMI 18.5-25 kg/m ^2^) US men and women are lean because they are practicing a healthy lifestyle (i.e., eating a healthy moderate calorie restricted diet, exercising regularly, and not smoking). This misuse of public and private funding, that for example paradoxically promotes high protein diets as a tool for treating obesity and the chronic diseases associated with obesity, has serious detrimental consequences on the environment, because intensive animal farming contributes to almost 20% of worldwide annual greenhouse gas emissions, to soil erosion, and pollution.
